# Navigating the Storm to Recovery through the Pictorial Representations of Persons in the Recovery Phase from Unipolar Depression

**DOI:** 10.3390/ijerph192013426

**Published:** 2022-10-18

**Authors:** Josianne Scerri, Amy Bonnici

**Affiliations:** 1 Department of Mental Health, Faculty of Health Sciences, University of Malta, MSD2080 Msida, Malta; 2 Faculty of Health, Science, Social Care and Education, Kingston University, Surrey KT2 7LB, UK; 3 Psychiatry Liaison Team, Mater Dei Hospital, MSD2090 Msida, Malta

**Keywords:** depression, interpretative phenomenological analysis, drawings, visual methods, qualitative

## Abstract

Depression is a highly complex mental illness that presents challenges, such as difficulties for persons with depression to communicate their experiences. This is compounded further by a paucity of in-depth and pictorial accounts on their experiences of the recovery process. The combination of pictorial representations and interviews with persons who are recovering from depression, may assist them in communicating these lived experiences. Five participants recovering from unipolar depression and who were in the late stages of recovery were recruited through purposive sampling. Data were collected through the conduction of art sessions, where participants pictorially represented their experience of living with depression and their road to recovery. Semi-structured interviews were then used to explore their artwork. The transcripts were analysed using Interpretative Phenomenological Analysis (IPA). Two superordinate themes emerged from participants’ interviews, namely: ‘A New Me in Me’ that incorporating changes in their identity, physical, emotional, and social experiences, and ‘Life as an amalgamation of colour’ describing their search for meaning and the importance of spirituality, hope, gaining control and positivity in the recovery process. The use of pictorial representations combined with interviews can add depth to participant narratives, that serve to enhance the therapeutic alliance between the patient–professional dyad.

## 1. Introduction

Depression is a leading cause of disability worldwide and a major cause of suicidal deaths with about 800,000 suicides per year [1]. It is estimated that the total number of persons with depression exceeded 300 million, hence confirming it as a significant global burden [1]. According to the Diagnostic and Statistical Manual of Mental Disorders (DSM V) depression is characterised by various physical and emotional symptoms, amongst which: a significant weight loss associated with a decreased appetite or a significant weight gain; recurrent thoughts of death; presence of anhedonia or a decreased pleasure in activities previously perceived as enjoyable; anergia, fatigue and exhaustion; excessive guilt; pervasive depressed mood for most of the day; feeling worthless and a decreased ability to focus and concentrate. For a diagnosis of depression to be confirmed, one of the symptoms must be depressed mood or anhedonia and at least five of the previously described symptoms must persist for over a minimum period of two weeks [2].

Current conventional treatments for depression include psychosocial support, antidepressant medications and different forms of psychotherapy [1]. Art and creativity have also been identified as key features in a person’s journey to recovery [3], where personal strengths, opportunities for change, perceptions, and emotional difficulties can be explored [4]. This association between creativity and recovery appears to be strongest for mood disorders, such as bipolar and unipolar depression [4]. Furthermore, empirical evidence posited by the American Psychiatric Association [5] recommends the use of psychodynamic therapy (that includes psychodynamic art therapy) as supplementary to the use of conventional treatments such as pharmacotherapy and psychotherapy for the treatment of depression. Despite this growing evidence, that highlights the applicability of art therapies, the former remain an under-utilised treatment for depression [6].

To date, various studies have explored the experiences of persons having depression through photo-elicitation [7]; visual metaphors relating to depression [8,9]; commonly presented images associated with depression [10]; and the use of art therapy in treating depression [3,6]. The aim of the qualitative study by Spandler et al. [3] was to explore the impact of art provision in facilitating recovery in mental health. The study incorporated an arts project with 34 participants experiencing mainly depression and anxiety. The findings demonstrated that such projects facilitate the creation of a sense of meaning and purpose; a rebuilding of identities; a fostering of hope and the development of new coping strategies (e.g., ‘grounding’ themselves by focusing on something absorbing) in participants. The study by Zubala et al. [6] consisted of a single group pre-test, post-test, follow-up study with five participants having mild to moderate depression. Group art therapy sessions were conducted twice weekly over 9 weeks and the participants’ responses to therapy were evaluated through questionnaires and interviews. The group sessions were described as enhancing motivation, creativity and spontaneity, a greater acceptance of depression and their own feelings and an increased openness towards others. The present study aims to extend the extant literature by exploring the lived experiences of a homogenous sample of participants, namely persons specifically living with unipolar depression and who are at a particular stage in their illness trajectory, namely in the late recovery phase of depression. The decision to focus on a homogenous sample was influenced by the fact that multifaceted characteristics relating to people, care processes and the contextual environment may impact the lived experiences and the narratives of these patients [11]. 

## 2. Materials and Methods

### 2.1. Research Design

The qualitative phenomenological research design used was Interpretative Phenomenological Analysis (IPA). This design was selected as it enables a deep exploration and understanding of the participants’ lived experience of a phenomenon [12].

IPA is based on three philosophical pillars of knowledge, namely phenomenology, hermeneutics and idiography [12]. Phenomenology incorporates exploring the subjective experience of how individuals make sense of their own life experiences. It contributes to health care research as it enables the exploration of a person’s subjective experience of a disease. The second pillar, hermeneutics incorporates the interpretative process with the researcher “making sense of the participant, who is making sense of x” [12] (p. 35). This is known as ‘double hermeneutics’ and involves the participant first interpreting their own experience and their own personal world, followed by the researcher who then strives to interpret the meaning of the participant’s account. Whilst idiography addresses the importance of an in-depth understanding of the unique experiences of the individual, within a specific context. Hence, an idiographic enquiry requires a homogeneous sample of participants who have experienced the phenomenon. In the present study, the participants represented a relatively homogenous sample, as they were all adult persons who were in the late recovery phase of depression and who were receiving psychiatric support.

### 2.2. Participants

Five participants were recruited for this study, of which three were males and two were females. These participants were selected through purposive sampling that enabled the selection of participants who could provide in-depth information relating to the aim of the study [13]. All individuals had a diagnosis of unipolar depression confirmed by a professional assessment and were in the last stages of recovery due to their imminent discharge from the ward. All the participants were at a stage where they could function independently, however two participants occasionally experienced low mood. Their ages ranged between 26 to 55 years, with a mean age of 40.6 years (SD = 9.93). The mean number of months since their diagnosis with depression was 97.20 (SD = 79.5) with a range between 8–240 months.

### 2.3. Data Collection

Participants were provided with diverse artistic tools and equipment that they could choose from. These included: paints, pencil colours, markers, pencils, clay, paper of varying size and craft supplies. Prior to the commencement of the art session, participants were given the time to experiment with the tools provided. Two participants required just a single prompt to start creating their artwork, whilst the other three required more frequent assistance and prompts. The psychodynamic art therapist asked participants about the colours that came to mind when thinking about their lived experiences of depression and the recovery process. This facilitated and encouraged the participants’ creative process. The sessions were held individually in a quiet room within the psychiatric hospital. Prior to commencement of the session, the staff were requested not to disturb the participant unless necessary. Each art session took approximately 1 h to complete.

Following the art session, participants were provided with a short break prior to the commencement of a semi-structured interview. The interview consisted of open-ended questions, specifically designed to elicit rich data on the impact of living with depression. The suitability of these questions was confirmed following feedback received from mental health professionals and previous service users of mental health services, The first question (i.e., can you tell me a bit about yourself?) served to provide contextual data of each participant, as well as serving as an ice breaker. The next question explored the participants experiences of depression and the recovery process namely: ’can you describe your experience of living with depression and the recovery process?’. The following questions namely: ‘can you describe your experience of today’s art session?; can you describe your artwork?; why did you choose the particular subject of this artwork and what meaning does this artwork hold for you?’ delved into the symbolism and imagery associated with depression and recovery used by the participants during the prior art session. The final question, ‘is there anything that you would like to add?’ provided participants with an opportunity to express any additional views, before the termination of the interview. Interviews ranged between 35 to 55 min long. Each interview was audio-recorded for transcribing and analysing at a later stage.

### 2.4. Procedure

Participants were recruited via two experienced mental health nurses (intermediaries) working within an acute psychiatric setting. These intermediaries were instructed to approach potential participants who fit the inclusion criteria provided. This procedure avoided feelings of coercion to participate in this study. Information letters were provided to the potential participants who were provided with sufficient time to decide on whether to participate. The service of a clinical psychologist was also available should any participant experience distress, however this service was not availed of. Participant names were also replaced by pseudonyms to safeguard the participants’ confidentiality.

### 2.5. Data Analysis

The data analytic process comprised an initial reading and re-reading of the transcripts and listening to the audio-recording for each transcript at least once. This enabled the researcher to actively engage with the original data. This was followed by initial note taking-in the left-hand margin of the transcript, where the analyst listed anything of interest in the transcript. This could relate to descriptive comments such as aspects that mattered to the participant (e.g., relationships) and/or even the language used to express their views. Developing emergent themes were then produced incorporating what is important in various exploratory statements, that were then grouped together. A searching for connections across emergent themes was then undertaken to determine how emergent themes related to each other. Those themes that represented similar understandings were clustered together beneath a single super-ordinate heading. The finalised list consisting of a superordinate theme and themes, was then formulated into a narrative account. This included a substantial number of verbatim extracts from the participants’ interviews to support each theme.

The trustworthiness and rigour of the study were also ensured using the four criteria posited by Lincoln and Guba [14] namely credibility, transferability, dependability, and confirmability. Credibility refers to confidence in the truth or quality of the data. This was ensured by explaining how the data were collected and analysed. Additionally, excerpts were provided that supported each theme enabling the reader to understand how the interpretation of data was conducted. Transferability refers to the extent to which study findings can be transferred or generalised to other groups or settings. In the present study, the findings generated relate to adult persons who were in the later stages of the recovery phase from depression. The domain dependability incorporates demonstrating that findings were consistent and can be repeated. Hence, the researcher provided a detailed description and rationale for every step of the research process. Whilst the final domain ‘confirmability’ describes the extent to which research findings represent data provided by the study participants rather than the biases of the researcher. The provision of open-ended questions in the interview schedule enabled the participants to voice their own experiences. Additionally, a reflexivity diary was kept, ensuring that the researchers were aware of any personal biases.

## 3. Results

The superordinate theme ‘Navigating the storm to Recovery’ encompasses two themes. The first theme ‘The New Me in Me’ describes the impact of living with depression, whilst the second theme ‘Life as an amalgamation of colour’ describes the participants’ experiences during the recovery process. The following table (Table 1) presents the themes and corresponding subthemes extracted from the data analysis.

### 3.1. The New Me in Me

**Figure 1:**
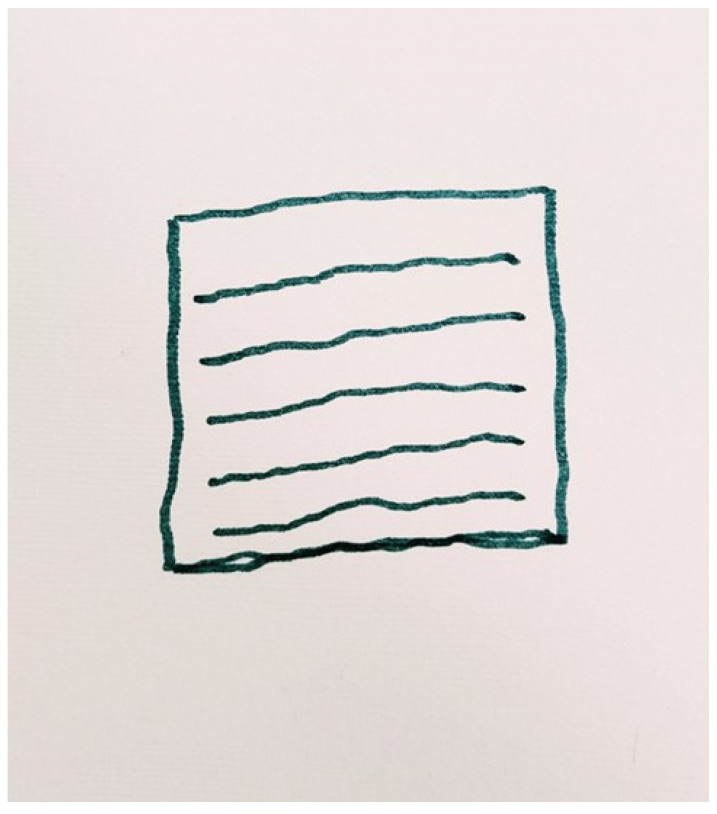
Associating Depression with a flatlined ECG (Clara’s representation).

Most participants described their experience of depression as being physically alive but void of any emotions and interest in life. Clara represented her experience of depression as that of a flatlining ECG (Figure 1). With this image, she acknowledged that although being biologically alive, her ‘true self’ incorporating a person with feelings and an interest in life no longer existed. Her ‘true self’ had died when she was diagnosed with depression.

*“I mean, in the sense that if you don’t participate in life, contribute, if you don’t socialise… what is there? You might be alive physically but not emotionally… it’s flatlining in the sense that the heart has stopped, life has stopped. From that point of view, so it’s not a death wish. Life as I knew it, and as I wanted it to be, had stopped.” Clara, p. 7, lines 1–6.*

Clara further expressed that everything in her life had lost its importance. This included a lack of interest in her own personal needs, hygiene, and her job that previously was key to her identity and self-worth. She further found it challenging to deal with any pending items, allowing them to accumulate and without having any plan to tackle them.

*“I stayed in bed for a whole month, I was so exhausted I didn’t wash, I hardly ate, maybe every five days or something like that…but nothing was important to me anymore. And my laptop, well, my work laptop, the battery has gone completely, so I haven’t even checked the many thousands of emails that are probably in… It’s just another thing that needs to be done, sometime in the future” Clara, p. 5, lines 49–54.*

Conversely, whilst Clara experienced a decreased appetite and weight loss, Daniela turned to food as a source of comfort. This behaviour led Daniela to gain further weight, with the resultant negative repercussions on her already fragile self-esteem. Daniela found herself immersed in a vicious cycle of weight gain and guilt that worsened her depression.

*“So, with the depression, obviously, I was comforting myself a lot with food, and I gained fifteen kilos. I mean, now I’ve stopped and I’m feeling good but it’s going to be difficult to lose that weight now. And like suddenly, a bunch of my clothes weren’t fitting me anymore…it’s hard. And it’s hard to love myself from the outside sometimes” Daniela, p. 3, lines 41–46.*

**Figure 2:**
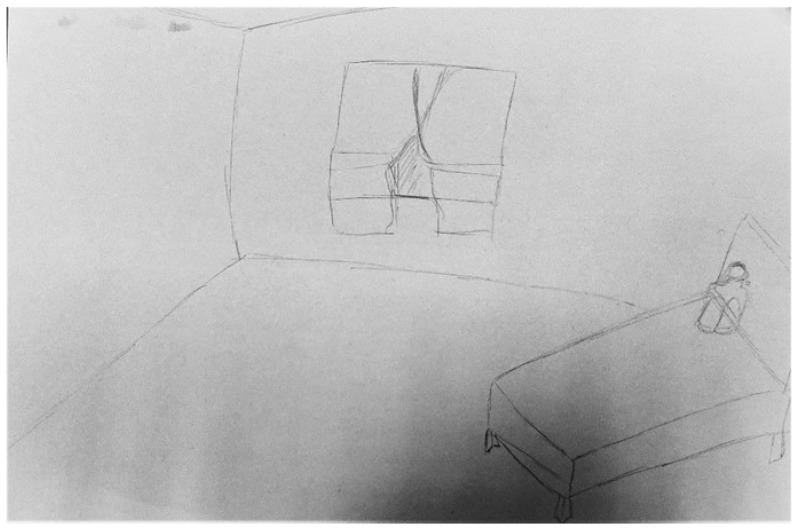
An insignificant, small figure in a large room (Daniela’s representation).

All the participants emphasised that depression converted them into an insignificant and insecure version of their former selves (i.e., the new me in me). Daniela confided that whilst struggling with depression she felt small, vulnerable, and insignificant. This was represented in her artwork with the image of a small figure, curled up in a foetal position on a large bed, within an equally large room (Figure 2). The large bare room provided an area of containment, whilst the foetal position presented a pictorial representation of how small, overwhelmed, and vulnerable she felt.

*“You feel so insignificant and so small that you don’t even want to take up any space in the room. Even though there is ample space, you just want to be small” Daniela, p, 6, lines 32–34.*

**Figure 3:**
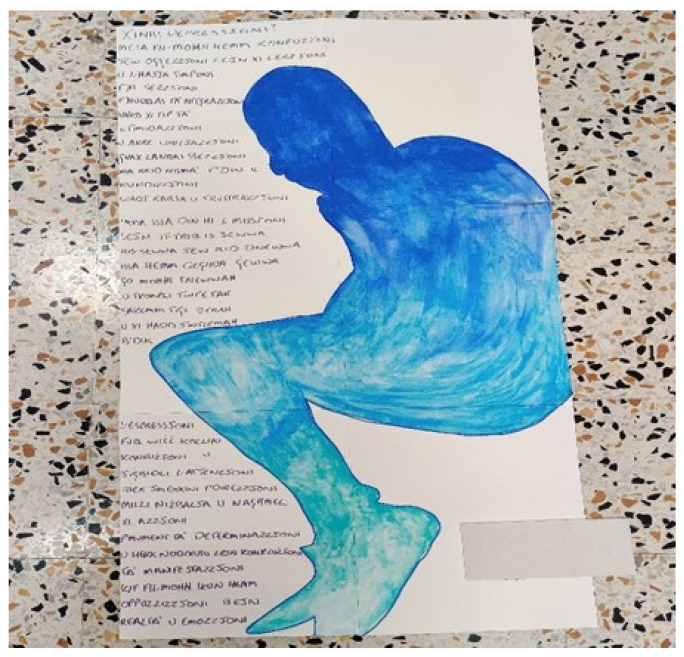
Curled up in a Foetal Position (Adam’s Representation).

Another participant (Adam) also presented the foetal position in his drawing. However, due to his interest in creative writing he surrounded the figure with words having a repetitive monotonous rhyming sequence such as depression, intimidation, objection, confusion, mission, section, integration, expression (Figure 3). Adam explained the presence of “*a lot of the same rhyme, you know so it’s flatlining. It flatlines like the depression” (Adam, p.6, lines 26–28)*. He further explained that the foetal position could be interpreted from a dual perspective. It represented his desire to feel safe and cared for just like a foetus enclosed within the mother’s womb. However, this enclosure additionally provided Adam with the isolation and social withdrawal that he yearned for. Adam further expressed that he used the colour blue in his artwork as depression relates to the expression of ‘feeling blue’. Whilst the varying shades of blue represented different degrees of severity of depression, with some days where the sadness, the ‘blue’ was worse and darker than other days.

This need to self-isolate was further elaborated upon by Daniela. She perceived herself as a drain on the mental energy of others. Daniela linked her view of a ‘depressed person’ with ‘bad company’. She rationalised that since she could not enjoy her own company, it was unfair to place that burden on others. Due to these circumstances, she kept others at a distance.

*“I wouldn’t put any effort into my friendships, so eventually you start to push people away. How long will people continue to message you if you continue to give them abrupt answers? …if I saw that someone doesn’t want to speak to me, I would try maybe once or twice and that’s all. I understand them one hundred percent. It’s not nice having the company of a depressed person. It isn’t…I didn’t even like my own company, so other people didn’t like my company either.” Daniela, p. 2, lines 27–34.*

The association between an acute change in emotional state and an upcoming episode of depression was further highlighted by Karl. He described becoming short-tempered and easily irritated. This left him feeling guilty about his reactions, as he was transformed into a very different person.

*“I would start to feel angry, and I would begin to snap at people who spoke to me. I always feel very sad and guilty after. Even when I raise my voice a little bit, that’s not me. I tend to be like a doormat, and everyone treads over me.” Karl, p. 3, lines 2–4.*

**Figure 4:**
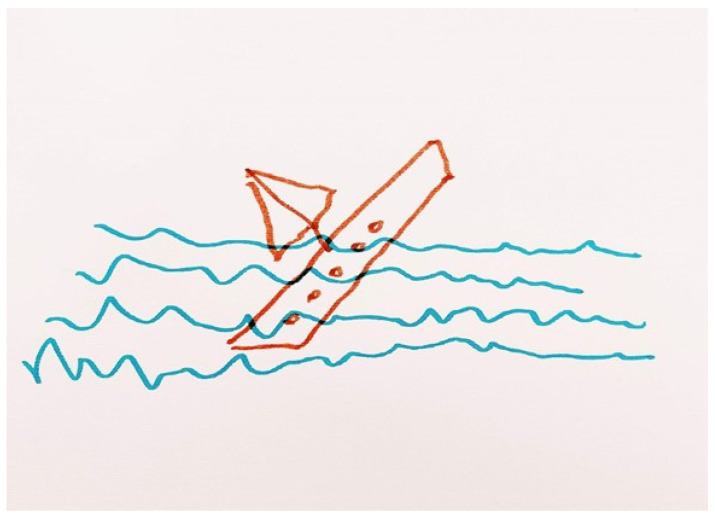
A sinking boat (Clara’s Representation).

Participants also highlighted their inability to experience pleasure in previously enjoyable activities. They described feeling helpless, out of control and hopeless. Clara pictorially represented this as a boat sinking with sea waves crashing over it (Figure 4). The boat depicted Clara in a state of depression, helplessly being pulled downwards into the sea, instead of remaining afloat. Clara further emphasised that the experience of sinking was so overwhelming that she would *“not be able to sort this (the depressed state) out by myself. It was larger than me, than my existence.” Clara, p. 6, 52–53*.

Steven further elaborated on the aspect of ‘sinking’ by contrasting his ‘prior’ self (i.e., a person who could face and overcome challenges) to his current self, a person lacking the skills to ‘fight’ depression. He further questioned how a person could ‘fight’ depression as it related to a mental rather than a physical phenomenon.

*“But now, I feel that I’m sinking fast because…I don’t have the skills (to fight the depression). If I had the skills, I would fight. But as it is I question… I’m fighting against what?” Steven, p.4, lines 3–5.*

Thoughts of death and dying also constituted a significant part of the participants’ experience with depression. Adam explained that these thoughts did not necessarily mean that he truly wished to die. However, they originated from the engulfing sense of despair and hopelessness at a life that was too burdened to bear.

*“I have had suicidal thoughts and those are the moments of determination when I say, you know XX (expletive language), I don’t care about life anymore and this is what I want to do now with my life, just waste it away” Adam, p. 8, lines 36–38.*

### 3.2. Life as an Amalgamation of Colour

**Figure 5:**
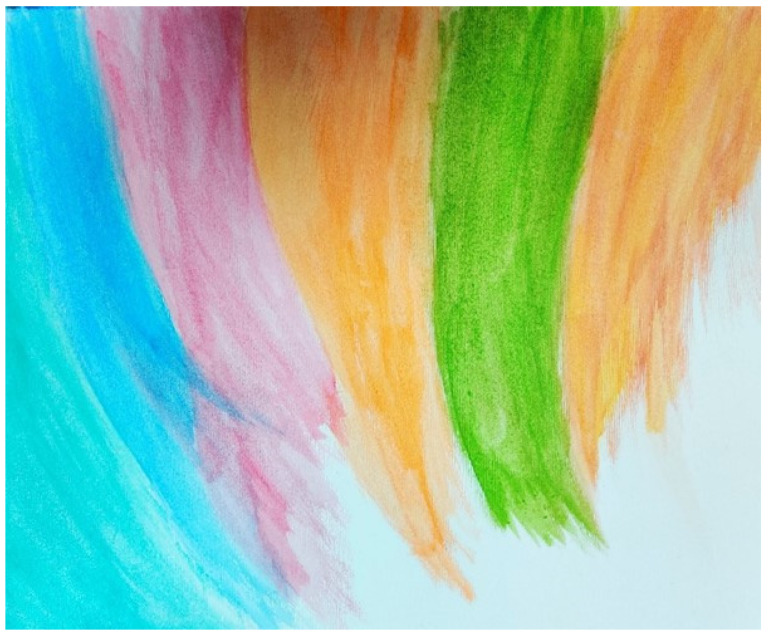
Recovery an amalgamation of colour: Daniela’s Representation.

Two participants (Daniela and Karl) both referred frequently to their search for meaning during the recovery process. Both participants were further advanced in the recovery process and expressed hope for a better future. Daniela depicted her recovery as an ‘amalgamation of colour’ (Figure 5) that filled the whole paper and should be contrasted to the dark colour used to depict her experience of depression (Figure 2). Daniela had begun living again and her life was imbued with meaning, feelings, and colour. Daniela also explained that she purposely left an unfinished area of white in her painting to signify uncertainty regarding what the future holds.

Daniela further elaborated on her search for meaning during the recovery process. She explained that there must be a reason that she was still alive, following unsuccessful attempts at terminating her life.

*“I tried taking a lot of pills (to terminate her life) …it did not work and I’m happy it didn’t because… Even though throughout the years, up until a few months ago I still had those thoughts, but I knew that since it didn’t work that time, that I’m still alive for some reason. I’m still trying to figure out the reason but, like, I know I’m still here for a reason” Daniela, p. 4, lines 46–50.*

**Figure 6:**
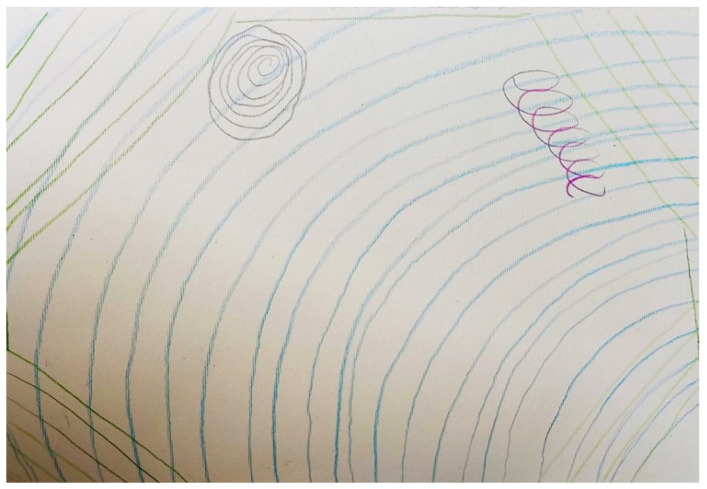
The field as a safe haven from challenges (Karl’s Representation).

Karl’s pictorial representation of recovery (Figure 6) consisted of repetitive green lines criss-crossing over the blue ones, creating a border around the page. The blue lines represented the ‘waves or challenges’ that he associated with having depression. However, the green lines represented his own safe haven, basically a field where he practiced gardening. This field provided him with a purpose, stability and control in a life that otherwise felt chaotic. Karl reported that he looked forward with enthusiasm to spending more time gardening in this natural setting.

*“It (the field) revolves around me and wraps me like a blanket. It’s one of the few places where I can do whatever I want. If I feel like planting seeds, I can do that, if I feel like doing something else, I can do whatever I want to” Karl, p. 6, lines 13–16.*

Karl further confided that he had grown to accept what God had permitted in his life (i.e., having experienced depression). He acknowledged that God would not place a greater burden on him than he could cope with. This gave him reassurance that he was not alone and that he really needed to strive to move forward.

*“Well, you must have a bit of courage to move forward. Not any special amount, but you must try to continue living with what God has given you” Karl, p. 3, lines 44–46.*

Karl further expanded on the aspect of ‘moving forward’ by emphasising the importance of maintaining an optimistic attitude.

*“If you don’t at least try to be positive, you cannot move forward. Even now, I’m going through some difficult things, but I try to focus on the positive because if I focus too much on the negative, I will end up in bed. The fact that I’m currently doing well with the medication and the TMS (Transcranial magnetic stimulation) means that I can cope with these difficult circumstances” Karl, p. 7, lines 33–39.*

## 4. Discussion

People who have experienced depression often describe the difficulty to communicate their experiences, such as feelings of emptiness and dysfunctional thoughts in words [7]. However, the use of imagery may assist in the communication of their experiences, whilst also providing experiential relief and cathartic expression [15].

### 4.1. Navigating the Storm to Recovery

According to Coll-Florit et al. [8] just over 25% of the metaphors used by persons with depression in their study related to the concept of the ‘split self’. This ‘split self’ personality was cited by all the participants in the present study, who expressed that whilst living with depression they were no longer their ‘true’ selves. This ‘depressed’ version of themselves was represented pictorially as a person lying in bed in the foetal position within a relatively bare room. The use of rooms conveys the sense of depression as a ‘captor’ (i.e., person staying inside), in addition to depression as ‘containment’ (i.e., held inside but within a safe space) [16]. Additionally, the representation of a foetal figure contributes to extant literature by exemplifying the participants yearning to feel safe and protected and disconnected from the outside world just like a foetus enclosed within the mother’s womb. By disconnecting from the outside world, a person with depression avoids the need to repress their emotions for fear of being judged and labelled as crazy or hysterical [7].

Daily activities were also described as a heavy burden. This was represented by one participant pictorially as a flat lined ECG. This represented a feeling of being emotionally dead, as participants failed to experience any enjoyment and satisfaction with life. This finding concurs with the research conducted by Hussain [7], where participants having depression struggled with undertaking daily tasks at school, the workplace and at home.

Changes in weight and the destructive impact on their fragile self-esteem were also expressed by two participants. Weight gain is identified as one of the many physical and biological symptoms of depression listed by the DSM V [1], yet it is scarcely mentioned in any of the critiqued or supplementary literature. Other biological symptoms associated with depression include an observable reduction in physical movement and fatigue [1]. In fact, fatigue was a common symptom expressed by all participants in the present study, who reported spending extended periods of time lying in bed. This finding corroborates that reported by Hussain [7] in which participants described sleeping for most of the day or lying on their couches.

Images of sinking or drowning also permeated throughout the participants’ artwork. These images represent the staggering feeling of hopelessness, loss of control and helplessness experienced by persons who perceive depression as unpredictable and uncontrollable. These ‘metaphors of descent’ or sinking are common in the narratives of persons with depression and are not an expression of weight or strain but rather a downward unrestrained movement within a physical space [17]. In fact, participants in a study by Charteris-Black [18] described descending into something deep, dark, and underground such as a hole or pit. Conversely, in the present study participants described descending into the sea, that represented a powerful, unpredictable, chaotic force that pulled the helpless person downwards. The constant referral to the sea in their narratives may relate to the sea being an integral part of their lives since the participants lived in an island state, but also possibly due to the rich use of metaphorical expressions in the Maltese language that associate struggles to a rough sea. This finding also implicates the importance of contextualising and interpreting personal narratives within their social and cultural context. The exploration of such narratives has further therapeutic implications where health care professionals ‘work’ with the patients’ beliefs and values. This involves gaining insight on the implicit messages conveyed in their language and the meaning that they associate to their life experiences [19]. Through an understanding of the persons values and beliefs, the professional can build up a picture of the person as a unique individual and integrate such information into a personalised care plan.

Another challenging consequence of depression is social isolation. One participant in Hussain’s study [7] described this as being on one side of a wall and trying to communicate with people on the other side. Such struggles to engage with others can arise due to various reasons, such as the person being too exhausted and/or lacking the skills to interact with others. Yet the present research contributes to extant literature by demonstrating that self-isolation may be maintained by persons with depression to safeguard others. For instance, Daniela perceived herself as a burden and interpreted that being in her company would mentally drain others.

Another metaphorical expression used by persons having depression relates to depression as an enemy to fight against. The term ‘fighting’ depression can be used in an empowering manner, such as targeting aspects that they disagreed with, such as the prejudice or stigma in their lives [8]. However, such war metaphors can also have a disempowering function such as when depression is perceived as an illness over which the individual has no control. In fact, one participant (Steven) questioned how can one ‘fight’ against depression that is linked to the mind, when one normally combats something physical in nature.

One participant (Daniela) created two pictorial representations in her art session–one representing her experience of depression and the other her experience of recovery. Yet, whilst her painting depicting depression is a pencil drawing entirely in grey, her artwork representing recovery is an abstract amalgamation of vivid colours. The colour grey in her artwork rather than simply relating to an absence of colour, corresponds to depression representing an absence of joy. This was also reflected in the children’s picture book ‘Misery Moo’ by Jeanne Willis and illustrated by Tony Ross [20], that deals with a depressive episode. In this book, the author describes the image of a grey and oppressive cloud that rains over the protagonist. The ‘greyness’ of the overall image evokes a dismal and disheartening feeling that mirrors what the depressed protagonist is going through [21].

Karl however expressed that his recovery was coupled with achieving some degree of control, meaning and stability in his life. For Karl this could be achieved through his passion of gardening that provided him with an element of control in his life, an antithesis to what depression represented for him. The impact of gardening on well-being was examined in a meta-analytic study by Soga et al. [22] that associated gardening with an increase in physical activity levels, cognitive function, quality of life, sense of community and reductions in depression and anxiety symptoms, mood disturbance and stress. Karl also further reiterated the importance of spirituality in his recovery process, that gave him a sense of hope when struggling with episodes of depression. In fact, religious beliefs may enable a more rapid recovery from depression through the provision of a sense of community and meaning to this experience [23]. This search for meaning serves as a buffer against threatening thoughts or situations [24], such as those experienced by persons living with depression.

### 4.2. Limitations

As typical of IPA research, the study was undertaken with a small sample of participants and hence research findings are not generalizable. However, the aim of this study was not to generalize the study findings but rather to elicit an in-depth narrative of the lived experiences of depression and the way these experiences can be depicted through artwork. It was also noted that some participants were far more naturally creative than others. This may have had implications on the artwork produced and hence, on the creation of a more authentic representation of their experience with depression.

## 5. Conclusions

Overall, this study contributes to the academic literature by exploring the use of art making to elicit in-depth participant experiences of unipolar depression and their journey towards recovery. Persons with depression often report the challenge of communicating their experiences especially with persons who do not have depression. Hence, through artwork and the sharing of personal experiences, care providers can explore, understand, and interpret the unique experiences of each person and in conjunction with the person living with depression, formulate a care plan that targets their needs and concerns.

The present study also highlights the importance of spiritual beliefs; the immersion in nature or pleasurable activities; a search for meaning; developing a sense of control and hope; as well as striving to be optimistic, as key to recovery. It also contributes to extant literature by highlighting the need to tackle misperceptions such as avoiding social contact with others to safeguard them. This can be targeted through therapies such as cognitive behavioural therapy. Additionally, it is important that health care workers reflect on the way that they communicate both verbally and non-verbally with persons experiencing unipolar depression, as the communication of stigmatising judgemental attitudes may have a negative impact on the fostering of a therapeutic alliance with the patient and consequently, their well-being.

## Figures and Tables

**Table 1:** Themes and subthemes representing participant experiences of depression and the recovery process.

Super-Ordinate Theme	Themes	Sub-Themes
Navigating the storm to recovery	The New me in me	- Emotional struggles - Social isolation - Loss of interest in personal hygiene and daily activities - Physical exhaustion and weight changes - Thoughts of death and dying.
	Life as an amalgamation of colour	- Searching for meaning - Immersing in nature - Spiritual beliefs - Focusing on the positive - Remaining hopeful

## Data Availability

All the relevant data are included in the manuscript.
